# Comprehensive evaluation of the use of technology in education – validation with a cohort of global open online learners

**DOI:** 10.1007/s10639-022-10986-w

**Published:** 2022-04-06

**Authors:** Jennifer W. M. Lai, John De Nobile, Matt Bower, Yvonne Breyer

**Affiliations:** 1 Macquarie School of Education, Macquarie University, Sydney, NSW 2109 Australia; 2 Macquarie Business School, Macquarie University, Sydney, NSW 2109 Australia

**Keywords:** Distance education and online learning, Applications in subject areas, Evaluation methodologies, Interactive learning environment, MOOCs, Confirmatory factor analysis

## Abstract

Although a large variety of methodologies, contexts and perspectives have been used to examine educational application of technology, there is a paucity of instruments that are designed to comprehensively evaluate the use of technology in education. This paper presents a Confirmatory Factor Analysis (CFA) of an instrument that incorporates eight key dimensions: learning, affective, behavioral, technology, design, pedagogy, presence/community, and institutional environment. These dimensions were derived from rigorous systematic literature review and field specialist validation processes. The model was then refined and empirically confirmed in this study by 1,352 participants undertaking a Coursera open online course. The results of applying the instrument, as well as qualitative feedback from participants, are shared to illustrate its breadth and utility. The final 28 item “Comprehensive Evaluation of Use of Technology in Education” instrument is provided in full to support consistent, holistic and robust evaluation and comparison of technology use across educational contexts.

## Introduction

Technology is now considered a critical and essential tool for enhancing teaching and learning by enabling students to access education from anywhere, at any time and often at their own pace (Turugare & Rudhumbu, 2020). With this increasing use of technology in education, practitioners and policy-makers need to deeply understand all aspects of how learning can be assisted with technology in order to enhance the overall student experience (Partala & Saari, 2015). Reflexive evaluation of technology use in education enables all stakeholders to understand the manifold impact of their approaches and continually refine their practices based on objective evidence. During the COVID-19 pandemic, the need for robust evaluation of technology use in education has only been heightened. The rapid and unplanned shift to online teaching caused by the *COVID-19* pandemic constitutes the largest disruption to education systems in history (Alqahtani & Rajkhan, 2020), with researchers arguing that there is an absence of comprehensive mechanisms to evaluate technology-enhanced learning (Al‐Taweel et al., 2021). Without robust, valid and comprehensive means of evaluating technology use during the switch to online learning, educational systems, institutions, and teachers may struggle to accurately gauge the impact of their changes or how to improve their approaches.

However, in the context of learning with technology, the evaluation process is complex and often controversial (Muller & Wulf, 2020). Phillips, Kennedy and McNaught observe that educational technology evaluation is multifaceted, involving the apprehension of a large number of interdependent variables (2012). Kirkwood and Price (2015) point out that during evaluation, researchers may focus on the technology itself rather than the way that the technology is used in the learning process. There are often methodological limitations in the way that the use of technology in education is evaluated, such as the construct and content validity of the measurement items, as well as the generalizability of the results (Cox & Marshall, 2007; Kirkwood & Price, 2015). Further, investigations about the use of technology are usually evaluated using a narrow perspective, with previous work by Lai & Bower (2019) showing that while a wide range of dimensions are evaluated throughout the educational technology literature (learning, affective elements, behaviors, technology, design, pedagogy, presence/community, as well as the institutional environment), research tends to typically focus on a small subset of these dimensions. To the best of our knowledge, there is no single comprehensive and robust model with which to evaluate the wide range of dimensions that influence technology-enhanced learning. This may be part of the reason that some researchers claim that there are not enough high quality evidence-based approaches to evaluate the impact of technology use in education (Kirkwood & Price, 2015). Additionally, there have historically been concerns about the exclusive use of either quantitative or qualitative research methods, that do not seize upon the power to adequately complement each other to enhance understanding of effects (Maxwell, 2016; Mertens & Hesse-Biber, 2013).

The development of an integrated model to comprehensively evaluate the use of technology in education can help educators plan, evaluate and execute learning technology in different contexts, make accurate comparison between approaches, positively reform curriculum, and make informed recommendations for educational policy. Researchers argue that there is widespread utility in adopting a comprehensive, rigorous, and multi-faceted method to evaluate technology use in education (Pickering et al., 2019; Reeves & Lin, 2020). However, the large number of interdependent variables involved in the evaluation of technology use in education (observed by Phillips et al., 2012), the complexity of evaluation (Muller & Wulf, 2020), and perennial issues surrounding content and construct validity (Kirkwood & Price, 2015) raise the question of whether it is in fact possible, within one instrument or approach, to comprehensively evaluate the use of technology in education. Accordingly and specifically, the research question of this study is: “*To what extent is it feasible to comprehensively evaluate the use of technology in education by using a single survey instrument?”* While there have been many course evaluation instruments that may incorporate technological aspects (e.g. Nicol et al., 2018; Nikolopoulou et al., 2020), this instrument *specifically evaluates the efficacy of technology use within courses* for the purposes of comparison and insights into technology-enhanced learning design.

The structure of this paper is as follows. First a background review is provided, that outlines existing instruments for evaluating technology use in education, as well as an overview of previous empirical work that we have conducted to determine the dimensions and items of import when evaluating the use of technology in education. An overview of previous work regarding MOOC evaluations is also provided, as background to the evaluation context that is used in this study. The methodology section explains the Confirmatory Factor Analysis (CFA) and the thematic analysis processes that were undertaken. The results of the CFA are then provided, along with the qualitative analysis of participant perceptions of the use of technology and the evaluation instrument. Critical reflections regarding the evaluation of technology in education follow in the final sections of the paper.

## Background

### Previous instruments for evaluating technology use in education

To evaluate the use of technology in educational contexts, scholars have used a wide variety of instruments to measure a range of different aspects within the learning environments being studied. Of course, learning is often evaluated in terms of the extent to which specific disciplinary outcomes are met, for instance as part of multimedia learning studies (e.g., Almasseri & AlHojailan, 2019; Kühl & Zander, 2017; Shamim, 2018). Additionally in terms of learning, researchers often examine the degree of cognitive load that is imposed in technology-mediated learning environments through instruments derived from the Paas (1992) Mental Effort Scale (e.g., Craig & Schroeder, 2017; Larmuseau et al., 2020; Wang & Antonenko, 2017). As another example of evaluating knowledge gains, scholars have used variations of the Technological Pedagogical Content Knowledge (TPACK) framework (Mishra & Koehler, 2006) to investigate teacher’s understanding relating to teaching with technology (Koh, 2020; Ozudogru & Ozudogru, 2019; Tondeur et al., 2020).

In addition to learning outcomes, the scholarly community has evaluated different dimensions of the application of technology in education in various ways. Regarding evaluating technologies themselves, the Technology Acceptance Model (Davis, 1989) has been applied in various instruments to investigate, for example, the perceived usefulness of MOOC (Alraimi et al., 2015), the perceived usefulness of eBooks (Jou et al., 2016) as well as the perceived ease of use and perceived usefulness on mobile library applications (Rafique et al., 2020). To examine the affective aspects of using educational technology, measurement items for instance the Motivated Strategies for Learning Questionnaire (Pintrich, Smith, Garcia, & McKeachie, 1991) were adapted by researchers to evaluate the values of use of clickers in classroom (Buil et al., 2016), as well as motivation of learners in three computer programming MOOCs (Alonso-Mencía et al., 2021). To evaluate the sense of presence encountered by respondents in various technology-enhanced learning situations, scholars have adopted the Community of Inquiry Framework developed by Garrison et al. (2000) to study blended synchronous learning environments (Szeto, 2015) and online feedback practices (Yang, 2016). Furthermore, to evaluate learning behaviors in technology-assisted learning, academics have used or modified the Online Self-Regulated Learning Questionnaire (OSLQ) (Barnard et al., 2009) to investigate the self-regulated behavior via mobile notifications and learning analytics (Tabuenca et al., 2015), and also the self-regulated learning in MOOCs in Russia (Martinez-Lopez et al., 2017).

However, each of the instruments and cases outlined in the examples above focuses on a narrow subset of dimensions to evaluate the use of technology in education, for instance, cognitive load, or learning outcomes, or motivation, or technology acceptance, or teacher knowledge, or presence, or self-regulatory behavior. Yet it is often important in educational evaluation and research to form a holistic view of how technology use impacts upon learning, rather than with relation to only one or a few dimensions of import. To the best of our knowledge there are no instruments that are used by researchers to examine the use of technology in education across a broad range of dimensions. Given the scope of the educational technology field, and its increasing importance in learning globally, there is a pressing need to develop a survey instrument to comprehensive evaluate educational technology use in education.

### Previous work regarding the evaluation of technology in education

In order to develop a robust and comprehensive instrument to evaluate the use of technology in the educational context, we first conducted a systematic literature review of how technology use in education has been evaluated, based on 365 papers published between 2015 and 2017 in *Computers & Education* (Lai & Bower, 2019). The analysis revealed that the evaluation of learning technology use tends to focus on eight themes: learning outcomes, affective elements, behaviors, design, technology elements, pedagogy, presence/community, and institutional environment. In addition, the analysis identified sub-dimensions of each dimension, which in turn formed the initial basis of items for each dimension in the survey instrument.

For instance, the systematic review found that *learning outcomes* includes the evaluation of performance, knowledge, achievement, or skills development like communication skills, interpersonal skills or motor skills (see studies for instance by El-Maghraby, 2021; Komalawardhana et al., 2021). *Affective elements* refers to learners’ perceptions, intentions, preferences, attitudes, values or beliefs (for example, see Hew et al., 2020; Sun et al., 2021). *Behavior* consists of interaction, participation, collaboration, and cooperation between or among learners (for instance, Bergdahl et al., 2020) whereas *design* comprises course quality, course content, course structure, resources or overall design (see Jahnke & Liebscher, 2020). *Technology* is usually measured by its perceived usefulness, perceived ease of use, functionality, or accessibility (for example Tang et al., 2020). *Teaching/pedagogy* includes pedagogical practice, teaching strategies or teaching quality (see, for example, Undheim & Jernes, 2020). *Presence/community* consists of social presence, co-presence or community as well as the presence in the environment (see Park & Song, 2020). *Institutional environment* considers the institutional capacity, institutional intervention, policy and support in facilitating the use of technology in teaching and learning (Huang et al., 2020).

Taken together, these dimensions are represented in several prevelant theoretical frameworks from within the technology-enhanced learning field, many of which have already been mentioned, including Cognitive Constructivism (Piaget, 1970), the Technology Acceptance Model (Davis, 1989), the Technological Pedagogical Content Knowledge (TPACK) framework (Mishra & Koehler, 2006), and the Community of Inquiry Framework (Garrison et al., 2000). The alignment between the eight dimensions and prevailing theory in the educational technology field has been extensively discussed in a separate paper ([Lai and Bower, paper currently prepared for submission]). While the eight dimensions were able to entirely encapsulate dimensions represented in prevailing theoretical frameworks, interestingly, none of the prevailing theoretical frameworks in the educational technology field contain all eight dimensions.

To further investigate and validate which dimensions and items were most important to consider when evaluating the use of technology in education, a field specialist validation was conducted (Lai et al., 2022). A total of 48 specialists in the educational technology research field were surveyed to determine their perceptions of the relevance of different constructs relating to the evaluation of technology use. There was an alignment between the constructs that field specialists felt were important and the eight dimensions in the systematic literature review, with 98% of field specialists agreeing that at least one item in each of the eight dimensions was relevant or highly relevant to the evaluation of technology in education.

The expert validation process, which also inquired as to the wording and clarity of the items included in the instrument, provided face and content validation for the survey instrument. Face and content validity are quality criteria when developing new measurement items in the educational technology field (Lin et al., 2016), and our sample of 48 field specialists used to undertake the face and content validation constituted one of the most rigorous instrument validation processes undertaken in the educational technology field (Lai et al., 2022).

However, face and content validity in and of itself is not suffice to establish the veracity of the survey instrument, because the items that had been composed to constitute each of the eight dimensions of evaluation of educational technology use may not in practice sufficiently represent those dimensions as factors. As such, a large-scale confirmatory factor analysis was conducted to establish whether or not the items designated to constitute the a priori factors did indeed sufficiently represent those factors. The context chosen to test and refine the instrument items was a Coursera Open Online Course.

### Massive Open Online Courses (MOOCs)

Massive Open Online Courses (MOOCs) have become popular due to their ability to provide high quality learning from almost anywhere, anytime (Al-Adwan, 2020). These courses have gained wide acceptance as a significant contribution to improving educational system quality and openness, with substantial growth of MOOC offerings during the COVID-19 pandemic (Impey, 2020). Scholars have investigated the success factors, best practices, and effectiveness of MOOCs (e.g., Albelbisi, 2020; Moreno-Marcos et al., 2018; Wu & Chen, 2017). For instance, assessment, pedagogy, technology, content, motivation, learner support and interactivity have been identified as factors that influence the effectiveness of MOOCs (Gamage et al., 2015). Loizzo et al. (2017) argue that a successful MOOC enables participants to gain knowledge and understand course materials, and success is linked to learners’ motivation and enjoyment of the MOOC and how applicable the knowledge gained is to everyday life. Reparaz et al. (2020) have argued that behavioral, cognitive and motivational factors affect the MOOC’s retention rate. Even though previous studies have investigated the effectiveness of MOOCs, the reliability and validity of results can be called into question, as there is a lack of a suitable tool to evaluate MOOCs themselves (Garreta-Domingo et al., 2018; Zhou, 2016).

A MOOC hosted on the Coursera learning platform was deemed a suitable context to test the validity of the items according to the a priori factor structure because, a) it constituted a public course that is openly accessible for inspection by people across the world (open research context), b) the survey could be completed by an international cohort of participants which in turn helps to establish the generalizability of the instrument (not subject to particular cultural or regional biases), c) MOOCs represent an area where suitable evaluation mechanisms are sought (as identified above), and d) the large sample of respondents that could be sourced (to increase reliability of the analysis). Note that while a MOOC was used as the context within which to conduct the confirmatory factor analysis, the proposed instrument was not in any way designed to specifically cater to MOOC evaluation. The intention of the instrument is to support evaluation of technology use in education for a wide variety of technologies and educational contexts. The opportunity to harvest qualitative feedback from participants about the MOOC and instrument is also seized, in line with recent momentum towards more mixed methods research (Hwang & Fu, 2019).

## Methods

As previously outlined, this study uses confirmatory factor analysis (CFA) to investigate whether a single instrument containing the eight evaluation dimensions identified in our previous review can reliably evaluate technology use in education. CFA evaluates a priori hypotheses and is largely driven by theory and is thus mainly used to verify the factor structure of a set of observed variables (Denovan et al., 2019). CFA analyses require the researcher to hypothesize in advance a number of factors, which was the case in this study where prior analysis had revealed eight categories of evaluation that occur in educational technology research. The CFA process is to test whether the hypothesized items/measures load onto the hypothesized factors (Tarhini et al., 2016).

Another possibility was to run an exploratory factor analysis (EFA), which is generally used when there is no pre-defined structure to the questions (Goretzko et al., 2019). This was not the case for this study, where the questions were arranged according to the eight themes identified through the literature review (learning, behavior, affective, etc.) as well as the corresponding sub-themes.

For thoroughness, an initial EFA was run to examine the data. Several different extractions, rotations and iteration boundaries were trialed. However, only three factors were obtained in total. Firstly, a small factor emerged containing six negatively worded items (e.g.*“The way the Coursera platform was used in this course did not enhance the design of the assessment tasks”*). Another factor contained five questions including items relating to interaction aspects (e.g. “*The way the Coursera platform was used in this course increased the amount I could interact with others”*). The remaining 23 items clumped together into one large factor containing items relating to the course in general, and the items to a certain extent measured the overall course impact of the students (for instance, “*The way the Coursera platform was used in this course helped me to perform better in this subject*”)*.* Accordingly, the research team proceeded with the CFA analysis, to determine whether the *apriori* factor structure incorporating a comprehensive array of dimensions derived from the literature could be validated. Numerous previous studies have conducted validation of questionnaires items using CFA alone, based on the literature as well as theoretical grounds (e.g. Burns et al., 2021; Rodríguez-Mantilla et al., 2019). The low of discriminatory power observed in the EFA is further discussed in the limitations section.

### Instruments

As explained in the Previous Work section (Sect. 2.2), the survey items were based directly on the eight dimensions and sub-elements that emerged from the previous systematic literature review published in *Computers & Education* (Lai & Bower, 2019). The exact wording of the questions is provided as part of the Results section.

Participants were asked to rate their levels of agreement with 38 items designed to measure the eight dimensions including learning outcomes, affective elements, behavior, design, technology, teaching/pedagogy, presence/community, and institutional environment on a 7-point Likert scale (0 = strongly disagree to 6 = strongly agree). The questionnaire combined both positively and negatively worded items to reduce the acquiescence bias that occurs when people agree with the statements without considering the actual contents (Podsakoff et al., 2003). All 38 items were arranged in random order to reduce the probability of bias based on contextual and compliant answering (Hills & Argyle, 2002). The term “*Coursera platform*” was used throughout all 38 items in the instrument to specify the technology being used and evaluated (for instance, “*The way the Coursera platform was used in this course helped me to learn more about the subject (LO1)*”; “*The way the Coursera platform was used in this course increased my participation (B1)*”.

Following the 38 Likert scale items, two open questions prompted participants to make any comments to clarify their responses above or express any other thoughts about using Coursera in learning Excel skills. Also, the participants were asked to suggest any improvements to the instrument questions.

**Fig. 1:**
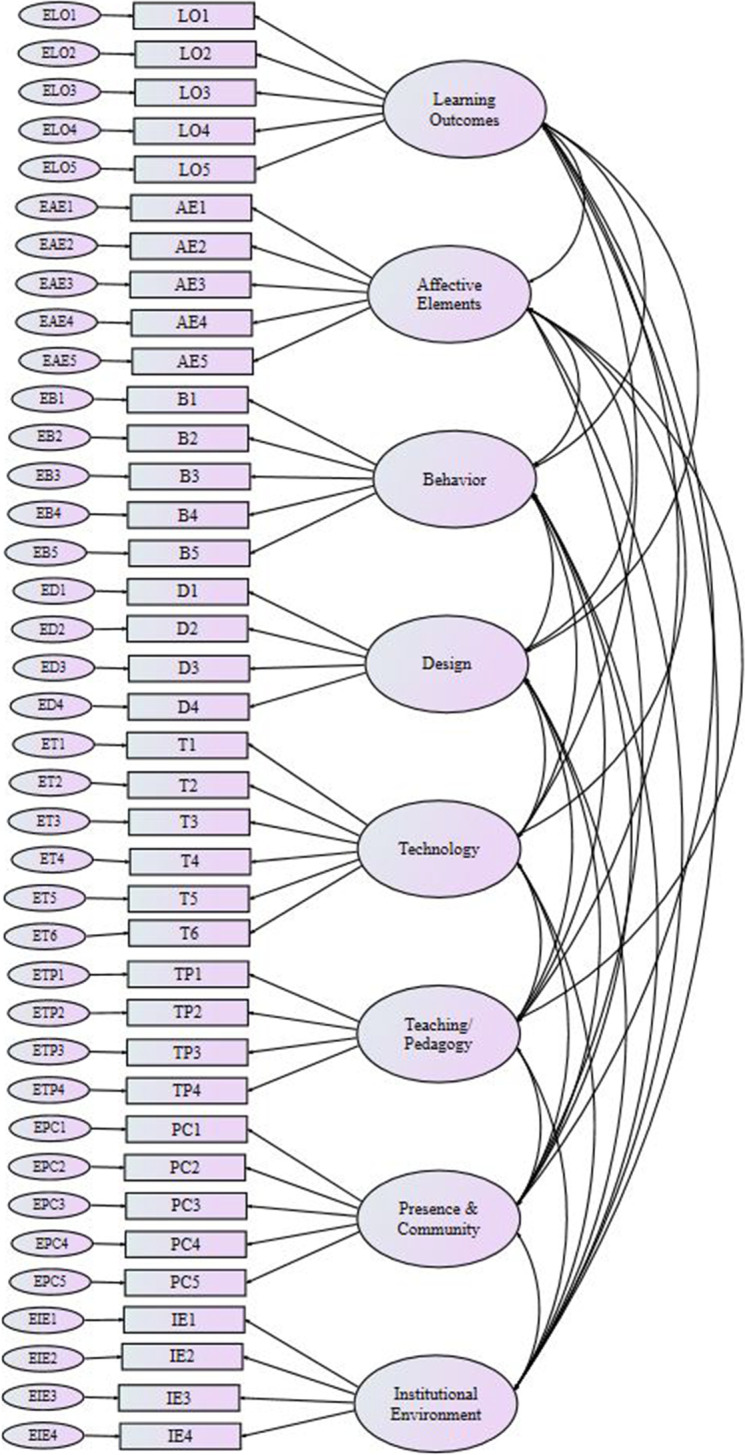
Hypothesized eight-factor structure model

The questionnaire also requested demographic data that were used to profile participants, including their gender, age, years of experience in using technology in the educational context, the hours per week spent on learning Excel skills with Coursera, the devices used to access the Excel course, and the number of different Coursera courses (on any subject) that they had done before this Excel course. Figure 1 shows the eight-factor model used to run the CFA.

### Data collection and participants

Following university ethical approval to conduct the study, participants were recruited from a MOOC hosted on Coursera. The MOOC, *Excel Skills for Business: Essentials* MOOC was originally created by an Australian University. The MOOC was not part of the University’s regular coursework, and rather was offered to participants from across the world as an open learning course. As explained in Sect. 2.3, the Coursera MOOC offered the advantages of providing an application of technology for evaluation, a publicly available research context, an international cohort and a large sample of respondents. There were over 200,000 enrolments in the Coursera MOOC at the time the study was conducted which was between February and July 2020. At the time, around 45,000 learners had completed the course. Among all learners, over 40% were aged between 25–34, 60% of them were male, and the learners were from different continents, especially Asia (e.g. India, Bangladesh, Philippines) and North America (e.g. USA, Canada, Mexico). Since the students were from a wide variety of regions with diverse demographic characteristics, they were to a certain extent representative of a large and diverse population, potentially enabling the results to be more generalizable to other settings.

The Qualtrics survey tool was used to collect all data, with the survey link distributed by placing it in both the Week 3 and Week 6 sections of the six-week MOOC for a total of 26 weeks. Open courses on Coursera do not have fixed offerings or semesters, and they are available at any time. Although data collection started at the beginning of the COVID-19 pandemic, there is no indication that COVID substantially impacted on either the responses of participants or the validity of the CFA. The course resources were already offered before the pandemic and were not changed to cater for the COVID-19 situation. While it was noted that enrollments were approximately double during the survey period, the rate of course completion was similar to the period before the pandemic and after survey collection.

In order to encourage honest responses, participants made contributions anonymously, and as an incentive to participate, the participants could follow a link to win an iPad or one of ten US$50 shopping vouchers. In total, 1,357 completed surveys were returned, of which 5 surveys were excluded because one of them did not indicate how many weeks he/she had spent completing the *Excel Skills for Business: Essentials* course, and the other four stated that they did not complete any week of the course. As a result, 1,352 responses were included in the study, a response rate of around 3%. A similar response rate was reported from other studies surveying MOOC learners from all over the world (Jung et al., 2019). Researchers recommend that a sample size greater than 200 is desirable to run CFA (DiStefano & Hess, 2005).

Among the participants, the majority were male (*n* = 857, 63.4%) with an average age of 26 years old. The demographics of the respondents were similar to the MOOC course in general, with slightly more male learners (63.4% as opposed to 60%) and the average age falling within the modal age range for the population of 25–34 years old. That is to say, the respondents to the survey appeared to broadly reflect the MOOC population being sampled. Also, this study was principally designed to provide a confirmatory validation of the instrument rather than evaluate the impact of the Coursera course, so any minor deviations between the respondent sample and course population were deemed acceptable.

The participants on average had 5.6 years of experience in using technology in educational contexts, and spent an average of 7.2 h per week learning Excel skills with Coursera. They had completed an average of 1.6 different Coursera courses (on any subject) before this Excel course, and most of them (68.1%) used a desktop/laptop computer to access the Excel MOOC course.

### Confirmatory factor analysis

Quantitative data from the Qualtrics questionnaire were analyzed using Statistical Package for the Social Sciences (SPSS), Version 27 and IBM SPSS AMOS 25. Before testing the model, the reliability coefficient for the instrument was calculated via Cronbach's Alpha. Cronbach’s Alpha is a generally agreed statistical measurement used for determining the internal reliability of the instrument (Taber, 2018).

The Comprehensive Evaluation of Use of Technology in Education instrument was tested by using an eight-factor structure model, with an initial corpus of 38 items. Model 1 consisted of all 38 items in the item pool. Byrne (2010) argues that when an initial model does not fit, researchers should improve the model based on modification indices and theoretical considerations. It is suggested that modification should cease if acceptable fit parameters are met. The deletion of the items was based on two criteria:

1. standardized factor loadings (Standardized regression weights) should be at least 0.5 and ideally 0.7 or higher (Hair et al., 2019);
2. the standardized residual covariances that are above 2.58 (Byrne, 2010).

The process of item reduction involved deleting items with the lowest factor loading first in accordance with Afthanorhan et al. (2014). In general, the process involved deleting one item at a time and then re-estimating the model.

Understanding critical practices in running CFA are essential for researchers interested in construct validation (Rodríguez-Santero et al., 2020), and in this study several recommended principles for conducting CFA were used. The constructs in this instrument were based upon the previous literature and the sample size (1,352) was at least adequate based on the number of items (DiStefano & Hess, 2005). During the item deletion process, the recommended threshold of the number of items per variable-ratio is suggested to be 3:1 (DiStefano & Hess, 2005), which was upheld in the current study. Further, a minimum of three items per scale is usually recommended, as this number will reliably yield convergent solutions in CFA (Marsh et al., 1998).

Maximum Likelihood was used to maximize the probability of a good model fit and has limited bias with large samples (Freedman, 2009). As numerous fit statistics consider different aspects of fit, it has been recommended that researchers should report multiple fit statistics in structural equation model (SEM) studies (Maydeu-Olivares et al., 2017). When evaluating the model fit, the overall chi-square statistic has often been used. Although this statistic has the advantage of having a known distribution, chi-square is heavily influenced by sample size, data nonnormality, and model complexity (Byrne, 2010). Therefore, several fit indices have been developed to remedy these problems. In this study, four indices were used to assess the degree to which the data fit the model: the ratio of chi-square to degree of freedom (χ2/df), the root mean square error of approximation (RMSEA), and the model comparison indices, namely the comparative fit index (CFI), Normed Fit Index (NFI), TLI (Tucker–Lewis index), and goodness of fit index (GFI). These indices are the most commonly used in complex models (e.g., DiStefano & Hess, 2005; Sternberg et al., 2001; Tarhini et al., 2016). Regarding the indices, Hu and Bentler (1999) suggest that a cut-off value close to 0.06 for RMSEA is acceptable. The other model comparison indices are reported between 0 and 1, with acceptable values being over 0.9 (Delcea et al., 2019; Peterson et al., 2006).

### Qualitative data analysis

The qualitative data for this research were analyzed using *NVivo*, Version 12. Open-ended survey responses are widely used to explore and understand participants' experiences and perspectives in a variety of ways, including for evaluative purposes (Vitouladiti, 2014). In this study, a thematic analysis of the open-ended responses to understand the perceptions of the participants of the Excel MOOC according to the eight dimensions of the model that had been previously established (learning, affective, behavioral, technology, design, pedagogy, presence/community, and institutional environment).

Following the procedures by Johnson and Christensen (2019), the initial codes were assigned according to the constructs of the eight-factor model, then we conducted a secondary coding phase to refine and consolidate different dimensions, and identify sub-themes. Thematic techniques were similarly used to analyse participant perceptions of the survey instrument itself. Quotes are used to provide primary evidence of participant perceptions and the themes raised by them. For example, qualitative data about learning outcomes covered aspects like the practicality of the knowledge (*The course gives me a grip on Excel reports and metrics)*. In addition, respondents offered favourable comments on design aspects, relating to the user-friendly interface (*It was an excellent platform*). Furthermore, positive affective aspects of the course were frequently mentioned (*It made home-based learning more fun and interesting instead of being a chore).* Further examples are provided in the Results section below. The outcomes of the thematic analysis were used to explain quantitative results through concurrent triangulation (Creswell, 2015), and additionally enabled the research team to garner insights into the perceived efficacy of the instrument items.

The Results section first reports the findings of the CFA. The CFA outcomes are then followed by descriptive statistics for each construct in the model, to illustrate how the instrument enables comparative evaluation across a range of dimensions. Then qualitative data is used to explain the underlying reasons for participant evaluations along the eight dimensions, showcasing how the qualitative components of the survey provide explanatory power to compliment the quantitative findings. Finally, an analysis of participants’ perceptions about the overall instrument design is provided, to inform further research and development.

## Results

The fit indices for all models used in the CFA process are shown in Table 1. Upon inspection of the 38-item 8-factor model (Model 1), the fit indices did not reach suggested thresholds (Hu & Bentler, 1999) (χ2/df = 11.860, NFI = 0.791, TLI = 0.785, CFI = 0.805, GFI = 0.661, RMSEA = 0.090). Item IE4 (“*The institution (Coursera Inc) did not provide the necessary infrastructure to facilitate the use of the Coursera platform in this course*”) was deleted from Model 1. Model 2, a 37-item model was rerun with slightly improved model fit (χ2/df = 10.551, NFI = 0.819, TLI = 0.815, CFI = 0.833, GFI = 0.712, RMSEA = 0.084). Since the figures did not meet the criteria for a reasonable model fit, another item—TP4 (“*The way the Coursera platform was used in this course did not enhance my impression of the teacher*”) was removed. The model was rerun with 36 items (Model 3) with the following model fit indices (χ2/df = 9.636, NFI = 0.840, TLI = 0.838, CFI = 0.854, GFI = 0.749, RMSEA = 0.080). More items were deleted according to the standardized factor loadings of the items in sequence B4 (“*The way the Coursera platform was used in this course reduced my ability to regulate my learning*”), PC4 (“*The way the Coursera platform was used in this course did not help me to feel part of a learning community*”), D4 (“*The way the Coursera platform was used in this course did not enhance the design of the assessment tasks*”), T5 (“*I had difficulties accessing the Coursera platform in this course*”), AE2 (“*The way the Coursera platform was used in this course made me feel more anxious about this subject*”), LO3 (“*The way the Coursera platform was used in this course increased the mental effort required to learn*”), B2 (“*The way the Coursera platform was used in this course increased the amount I could interact with others*”) and PC5 (“*The way the Coursera platform was used in this course enabled me to immerse myself in the learning environment*”). The last column in Table 1 also indicates the standardized factor loadings of the deleted items, and the factor loadings of the deleted items ranged from 0.101 to 0.558. After deleting 10 items, model fit was achieved (Model 11 – 28 items), as χ2/df = 3.672, NFI = 0.959, TLI = 0.964, CFI = 0.970, GFI = 0.940, RMSEA = 0.044).

**Table 1:** Fit indices for the proposed model

	CMIN	Df	χ2/df	NFI	TLI	CFI	GFI	RMSEA	Deleted	Estimates
Model 1—38 items	7555.135	637	11.860	0.791	0.785	0.805	0.661	0.090		
Model 2—37 items	6341.035	601	10.551	0.819	0.815	0.833	0.712	0.084	IE4	0.101
Model 3—36 items	5453.860	566	9.636	0.840	0.838	0.854	0.749	0.080	TP4	0.047
Model 4—35 items	4581.328	532	8.612	0.862	0.861	0.876	0.789	0.075	B4	0.060
Model 5—34 items	3826.540	499	7.668	0.882	0.883	0.896	0.812	0.070	PC4	0.063
Model 6—33 items	3161.749	467	6.770	0.901	0.903	0.914	0.836	0.065	D4	0.087
Model 7—32 items	2743.394	436	6.292	0.913	0.915	0.925	0.856	0.063	T5	0.091
Model 8—31 items	2466.739	406	6.076	0.921	0.923	0.933	0.871	0.061	AE2	0.122
Model 9—30 items	2284.884	377	6.061	0.926	0.927	0.937	0.879	0.061	LO3	0.351
Model 10—29 items	1660.065	349	4.757	0.944	0.948	0.955	0.913	0.053	B2	0.464
Model 11—28 items	1182.281	322	3.672	0.959	0.964	0.970	0.940	0.044	PC5	0.558

The research team also checked that there were reasonable theoretical grounds for deleting each of the items. On inspection, it was apparent that some deleted items did not have sufficient content validity with respect to the dimension they were measuring (for instance, in D4 assessment measuring design, or TP4 the impression of the teacher measuring teaching). In other cases, the deleted items potentially over-attributed the impact of technology use (for instance in B4 that the technology impacts on self-regulation, or AE2 that the technology was causing anxiety). Across all deleted items, there were sufficient theoretical grounds (either content validity or over-attribution) for deletion.

**Fig. 2:**
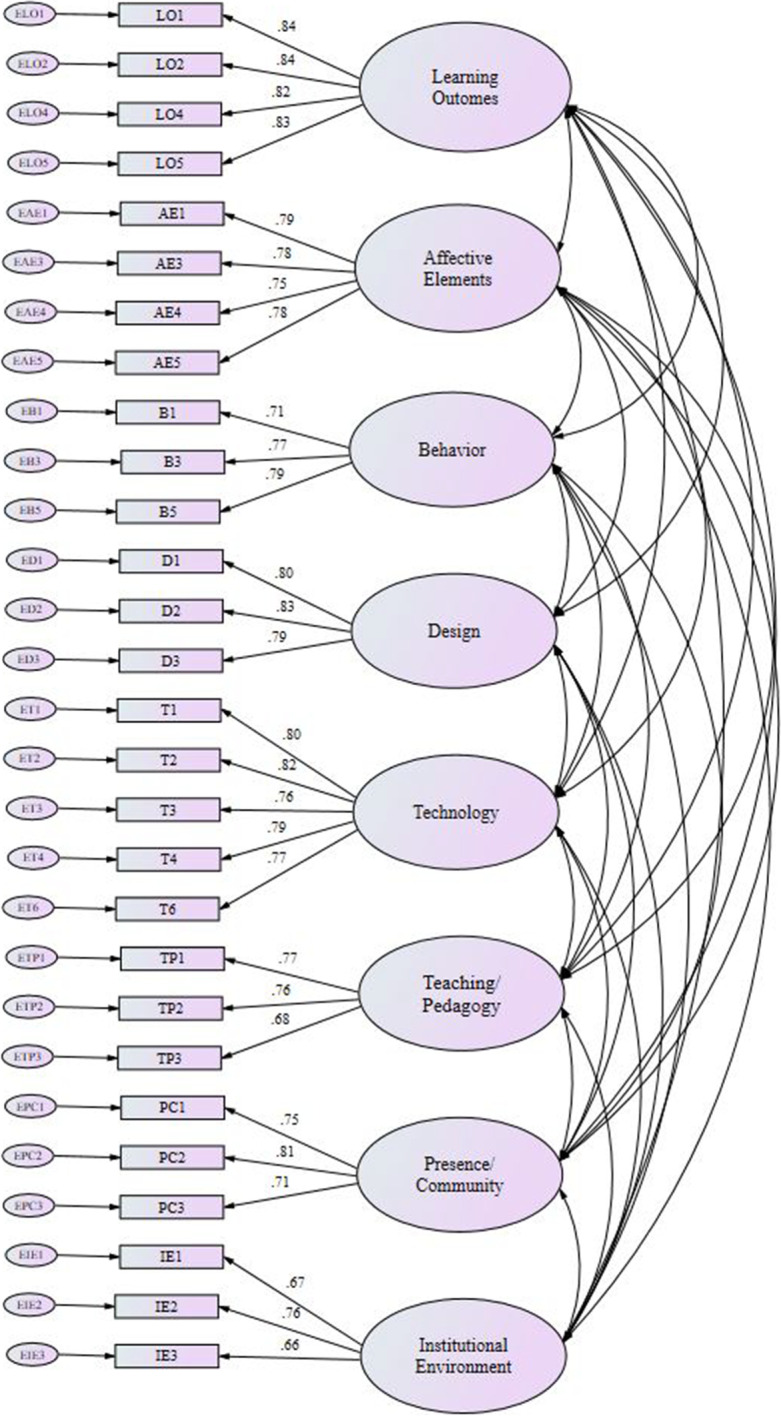
Standardized loading factor coefficients of the eight-factor structure model

The theoretical underpinning of all factors was supported, with the deletion of ten items to bring fit indices to acceptable levels. In addition, we recognize how important it is to balance the fit indices values and that the integrity of the model is not adversely affected by the modifications. The results achieved indicate that it was in fact possible to create a comprehensive instrument to evaluate the use of technology in this educational context. The conceptual model is outlined in Fig. 2 below.

In addition to model fit indices, factor loadings were examined. Table 2 shows the standardized loadings of all items in the model. Typically, standardized loading factor coefficient of 0.70 can immediately be regarded as valid (Gatignon, 2010) with studies sometimes accepting the standardized loading factor coefficient over 0.5 (Widodo et al., 2020). In this study the factor loadings for all items were in the range 0.66 to 0.84, which was regarded as acceptable.

**Table 2:** Item-factor standardized regression weights in the Comprehensive Evaluation of Use of Technology in Education (CEUTIE) model

Regression			Estimate
The way the Coursera platform was used in this course helped me to learn more about the subject (LO1)	⬅	Learning Outcomes	0.84
The way the Coursera platform was used in this course helped me to perform better in this subject (L	⬅	Learning Outcomes	0.84
The way the Coursera platform was used in this course helped me to perform better in this subject (LO2)	⬅	Learning Outcomes	0.82
The way the Coursera platform was used in this course has improved my level of knowledge in the subject area (LO5)	⬅	Learning Outcomes	0.83
The way the Coursera platform was used in this course enhanced my attitudes towards the subject (AE1)	⬅	Affective Elements	0.79
The way the Coursera platform was used in this course helped to improve my confidence in this subject (AE3)	⬅	Affective Elements	0.78
The way the Coursera platform was used in this course enhanced my motivation to learn (AE4)	⬅	Affective Elements	0.75
The way the Coursera platform was used in this course made learning more enjoyable (AE5)	⬅	Affective Elements	0.78
The way the Coursera platform was used in this course increased my participation (B1)	⬅	Behavior	0.71
The way the Coursera platform was used in this course increased my ability to reflect upon my learning (B3)	⬅	Behavior	0.77
The way the Coursera platform was used in this course enhanced my overall engagement (B5)	⬅	Behavior	0.79
The way the Coursera platform was used in this course enhanced the overall design of the subject (D1)	⬅	Design	0.8
The way the Coursera platform was used in this course enhanced the subject content (D2)	⬅	Design	0.83
The way the Coursera platform was used enhanced the course structure (D3)	⬅	Design	0.79
The Coursera platform used in this course was of high quality (T1)	⬅	Technology	0.8
The functionality of the Coursera platform used in this course helped me to learn the subject (T2)	⬅	Technology	0.82
The Coursera platform used for learning in this course was easy to use (T3)	⬅	Technology	0.76
The Coursera platform used for learning in this course was reliable (T4)	⬅	Technology	0.79
The Coursera platform was useful to support learning in this course (T6)	⬅	Technology	0.77
The way the Coursera platform was used in this course increased my overall perceptions of the teaching quality (TP1)	⬅	Teaching/Pedagogy	0.77
The way the Coursera platform was used in this course enhanced teaching (TP2)	⬅	Teaching/Pedagogy	0.76
The way the Coursera platform was used in this course enhanced feedback processes (TP3)	⬅	Teaching/Pedagogy	0.68
The way the Coursera platform was used in this course enhanced my sense of connection with the teacher (PC1)	⬅	Presence/Community	0.75
The way the Coursera platform was used in this course enhanced my sense of being present in the class (PC2)	⬅	Presence/Community	0.81
The way the Coursera platform was used in this course enhanced my sense of connection with other students (PC3)	⬅	Presence/Community	0.71
There was good technical support for the use of the Coursera platform in this course (IE1)	⬅	Institutional Environment	0.67
The institutional support provided by Coursera Inc for the use of the Coursera platform positively contributed to my learning experience (IE2)	⬅	Institutional Environment	0.76
The institution (Coursera Inc) embraces the use of the Coursera platform in education (IE3)	⬅	Institutional Environment	0.66

### Specific findings from the evaluation

#### Descriptive statistics

The final model was able to broadly reflect the participants’ evaluation of educational technology use within this (Excel MOOC) context. Participant ratings are shown in Table 3 below.

**Table 3:** Descriptive statistics of the final 28-item eight-factor model

	Mean	SD	Cronbach’s alpha
Dimension 1: Learning Outcomes	**5.34**	**0.89**	**0.90**
The way the Coursera platform was used in this course helped me to learn more about the subject (LO1)	5.33	1.03	
The way the Coursera platform was used in this course helped me to perform better in this subject (LO2)	5.28	1.03	
The way the Coursera platform was used in this course increased my skills in the subject area (LO4)	5.36	1.01	
The way the Coursera platform was used in this course has improved my level of knowledge in the subject area (LO5)	5.40	1.00	
Dimension 2: Affective Elements	**5.25**	**0.89**	**0.86**
The way the Coursera platform was used in this course enhanced my attitudes towards the subject (AE1)	5.18	1.05	
The way the Coursera platform was used in this course helped to improve my confidence in this subject (AE3)	5.34	1.02	
The way the Coursera platform was used in this course enhanced my motivation to learn (AE4)	5.22	1.08	
The way the Coursera platform was used in this course made learning more enjoyable (AE5)	5.25	1.10	
Dimension 3: Behavior	**5.07**	**0.98**	**0.80**
The way the Coursera platform was used in this course increased my participation (B1)	4.99	1.24	
The way the Coursera platform was used in this course increased my ability to reflect upon my learning (B3)	5.11	1.11	
The way the Coursera platform was used in this course enhanced my overall engagement (B5)	5.10	1.13	
Dimension 4: Design	**5.11**	**0.95**	**0.85**
The way the Coursera platform was used in this course enhanced the overall design of the subject (D1)	5.09	1.11	
The way the Coursera platform was used in this course enhanced the subject content (D2)	5.12	1.10	
The way the Coursera platform was used enhanced the course structure (D3)	5.12	1.05	
Dimension 5: Technology	**5.31**	**0.85**	**0.89**
The Coursera platform used in this course was of high quality (T1)	5.32	1.03	
The functionality of the Coursera platform used in this course helped me to learn the subject (T2)	5.28	1.04	
The Coursera platform used for learning in this course was easy to use (T3)	5.35	1.03	
The Coursera platform used for learning in this course was reliable (T4)	5.32	1.00	
The Coursera platform was useful to support learning in this course (T6)	5.28	1.02	
Dimension 6: Teaching/Pedagogy	**4.92**	**1.00**	**0.77**
The way the Coursera platform was used in this course increased my overall perceptions of the teaching quality (TP1)	5.04	1.11	
The way the Coursera platform was used in this course enhanced teaching (TP2)	5.01	1.15	
The way the Coursera platform was used in this course enhanced feedback processes (TP3)	4.70	1.34	
Dimension 7: Presence/Community	**4.30**	**1.34**	**0.80**
The way the Coursera platform was used in this course enhanced my sense of connection with the teacher (PC1)	4.59	1.49	
The way the Coursera platform was used in this course enhanced my sense of being present in the class (PC2)	4.48	1.50	
The way the Coursera platform was used in this course enhanced my sense of connection with other students (PC3)	3.80	1.76	
Dimension 8: Institutional Environment	**4.99**	**0.98**	**0.73**
There was good technical support for the use of the Coursera platform in this course (IE1)	4.85	1.28	
The institutional support provided by Coursera Inc for the use of the Coursera platform positively contributed to my learning experience (IE2)	5.10	1.15	
The institution (Coursera Inc) embraces the use of the Coursera platform in education (IE3)	5.02	1.22	

The descriptive statistics showed that the mean scores of all items ranged from 3.80 to 5.40, above the mid-point of 3 (see Table 3). These results showed that the majority of the participants expressed generally positive answers to the variables used in the research model. The standard deviations ranged from 1.00 to 1.76. In this study, all eight constructs had alpha values above 0.7. Hence the results indicated good internal consistency of items in the measurement scale (Hair et al., 2019).

**Fig. 3:**
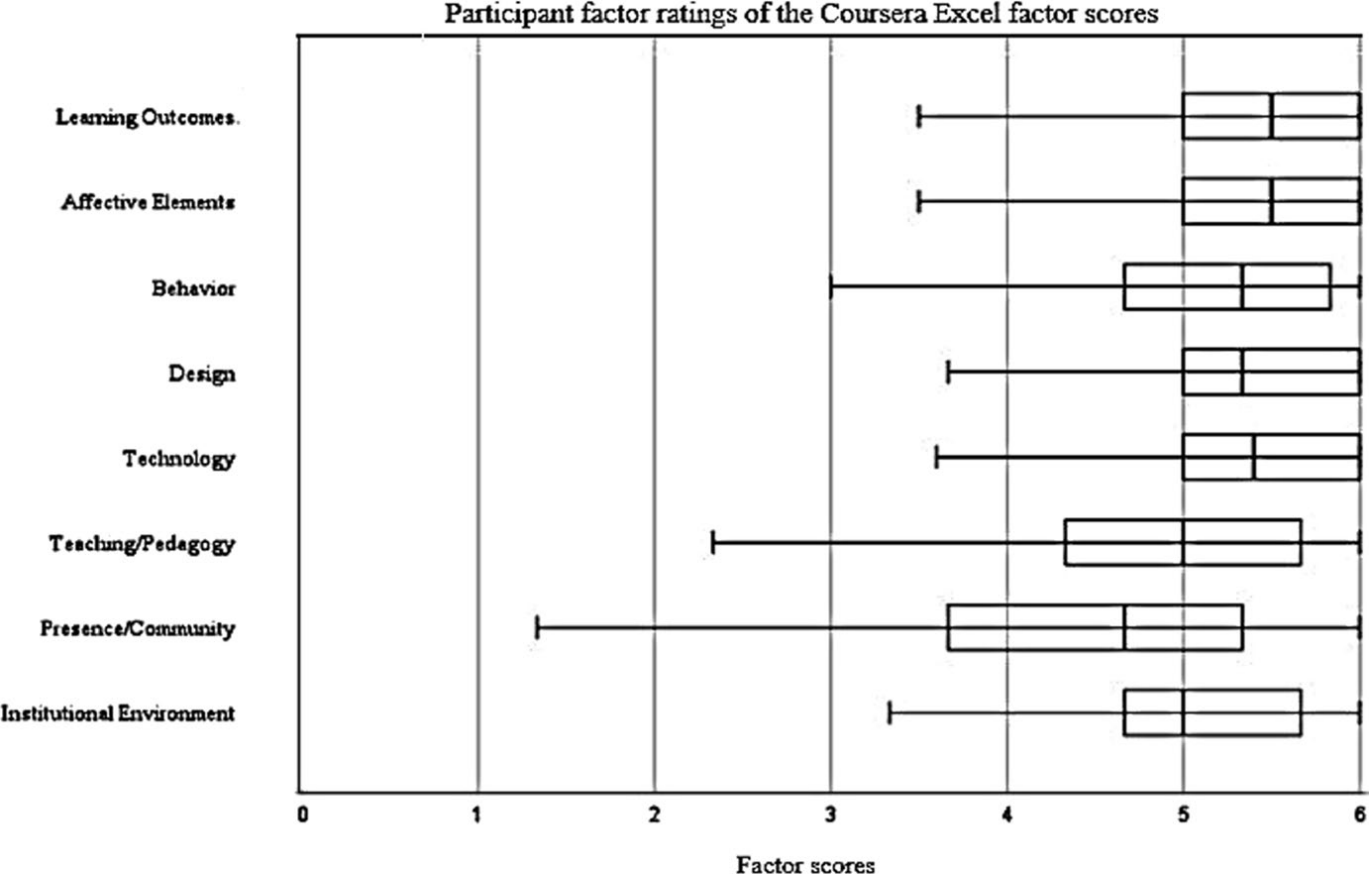
Participant factor ratings of the Coursera Excel factor scores for the Comprehensive Evaluation of the Use of Technology in Education model

Figure 3 shows a graphical (box plot) representation of participant feedback according to the eight constructs, which enables broad and clear evaluation of the technology using across a wide range of dimensions. The mean evaluation of the learning outcomes (M = 5.34) was the highest of any factor, while the lowest-rated factor was presence/community. There was the most variation in presence/community, whereas technology had the least variation. Thus, the instrument provides an immediate way to compare and contrast student perceptions of the technology use across a broad range of relevant constructs.

In addition, a multi-group analysis of the CFA model was performed across two groups (gender in this case) in order to check the variance of the scales (Yu et al., 2019). The resulting p-value of the comparison model was 0.203, indicating that there was no significant difference between the two CFA models. Hence, both the genders had similar response profiles.

#### Critical reflections from the participants

##### Participant qualitative feedback about the Coursera MOOC

Table 4 in Appendix provides illustrative participant responses to an open-ended question “Would you like to make any comments to clarify your responses above or express any other thoughts about using Coursera in learning Excel skills?”, with the responses able to be categorized into the eight dimensions of the model. The most frequently referenced dimension was affective elements (*n* = 448), followed by design (*n* = 363), learning outcomes (*n* = 325), technology (*n* = 238), teaching/pedagogy (*n* = 163), behavior (84), institutional environment (*n* = 45), and presence/community (*n* = 21).

The qualitative analysis of open-ended responses provided insights into the reasons underpinning the categorization of each of the eight dimensions, which constitutes a crucial aspect of the Comprehensive Evaluation of Technology in Education instrument. For instance, learning outcomes was rated the highest (average rating of 5.34/6) among all the eight dimensions, often because participants found the course contents were practical (*I applied this knowledge in a practical way. This course taught me the importance of Excel in professional life*), and helped them to improve Excel skills/knowledge (*Coursera topics on Learning Excel skills improves my knowledge in performing and creating graphs and shortcuts*). Participants also evaluated technology aspects favourably (5.31/6), often because of the usability of the platform (*The platform provided very clear instructions therefore was easy to use*). The positive evaluation of affective aspects of the MOOC (5.25/6) was largely due to participant perceptions of how the MOOC increased their motivation and confidence (*The structure and the methods that Coursera used are very easy and make me feel motivated; I am a Supervisor in a company, now I can easily make rosters, doing stocktake and I am confident in using excel).* Design had an average rating of 5.11/6 because participants liked how well the course was structured (*I liked the format of the course, how it was divided into weeks and how each week had the same structure).* Participants often evaluated behavior impact positively (5.07/6) because the course enabled them to self-regulate their learning (*Learning Excel skills through Coursera enabled me to complete the tasks at my own pace while keeping me on track by using the deadlines as a guide*). Institutional environment, support and policy were also mentioned by the participants but some participants were not satisfied with the institutional support (4.99/6) (*I do not have any email contact or something like that if I need help*). Teaching/Pedagogy was one of the most discussed aspects, but some participants would like the teaching staff to provide more illustrations when teaching (4.92/6) (*If you can be more explicit when teaching and bring in more examples or illustrations it will be very better*). The last dimension, namely presence/community was rated the lowest (4.30/6) as some participants did not have any sense of being part of the community (*I don't find there to be any sense of "community"—the usage of the community platform to ask questions is not very active as you can get responses after a month when the course period is already over*).

Unsurprisingly, there was considerable variance in comments relating to each dimension, and the examples above are used to illustrate the kinds of insights that can be acquired from these open-ended responses.

The qualitative component of the evaluation instrument provide critical explanatory power regarding the quantitative findings, which is in accordance with the general push towards mixed methods research in the educational technology field (Blayone et al., 2018). Additionally, all substantive comments in response to the open-ended questions could be categorized according to the eight evaluation dimensions, further demonstrating the efficacy of the eight factor model.

##### Participant feedback about the survey

Participants’ responses to Question “*Are there any ways that this survey could be improved”* received 1293 responses, with 445 being positive codes, whereas 414 were classified as negative aspects. The remaining codes were NA/None (see Table 5 in Appendix for illustrative quotes). Participants were overall satisfied with the survey (*n* = 237), and many indicated that no improvement was needed (*n* = 144) (*No improvements needed; It was really good and no need for improvements*). Specifically, they believed it was a comprehensive survey (*n* = 57) which covered all relevant aspects (*The survey was already able to cover all the necessary aspects; The survey was comprehensive*). The survey was also regarded as well-structured and well-designed (*n* = 21 codes) (*The survey is very well designed, no issues found; It was well structured*). Survey questions were easy to understand and direct (*n* = 16) (*The survey is short and easy to understand; The questions are asked directly*). To have received more positive responses than negative responses to a question asked for ways to improve the survey was unexpected.

Although participants comments about the survey were mostly positive, there were five main suggestions about the questionnaire items. Firstly, participants would like to see improvements in question wording (*n* = 118) (*I think the mixture of negative–positive statements is strategically placed but not all are proficient in English, so, it might be confusing or mixed up by others; Some questions seem to be quite similar*). Also questions could focus more on the in-depth understanding of different aspects, for instance, “*Probably discuss more Coursera specific features*”, “*how the participants are using the knowledge of this course*”, “*the survey could incorporate more questions about the interaction (type, quality, frequency) between people*”. The layout of the questionnaire could be improved with better visual presentation (*n* = 49) (*It will be improved by adding some graphical and image structure. It will look attractive; The up-down arrows are somehow irritating. Can use toggle button instead; This is good maybe color change here and there*). There were 43 relating to the reduction in the number of questions in the questionnaire (*I think the questions are too many and some of them look similar, so they can be omitted to reduce the survey time*). Furthermore, there were discrepancies regarding the grading scales of the items. Two of them recommended using the scale from 0–10 whereas the others preferred 3 to 4 rating scales (*n* = 42). Ways in which these comments have been considered and addressed in the final instrument are discussed in the next section, Discussion and Conclusion.

## Discussion and Conclusion

Evaluation of technology use in education typically focuses on a small number of dimensions at the expense of others. As far as we know, there is no single tool for the comprehensive evaluation of technology use in education across all eight dimensions that were observed as part of an earlier systematic literature review (learning, affective, behavioral, technology, design, pedagogy, presence/community, and institutional environment). Yet, any evaluation of technology use in education that omits one or more of these dimensions is at risk of missing important insights regarding the impact of the technologies applied. This study derived and validated 28 item Comprehensive Evaluation of the Use of Technology in Education (CEUTIE) instrument that can be used to comprehensively and reliably evaluate all eight dimensions of technology use in learning and teaching. The eight-factor model was validated by CFA results, with χ2/df = 3.932, NFI = 0.959, TLI = 0.964, CFI = 0.970, GFI = 0.940, RMSEA = 0.044).

For the *Coursera Excel for Business: Essentials* MOOC that was used as the context for the CFA, descriptive statistics showed that the mean scores of all items were above the mid-point 3, ranging from 3.80 to 5.40. These results indicated that the majority of the participants gave generally positive answers to the variables used in the research model. In addition, all eight constructs had alpha values above 0.7, thus the results indicated good internal consistency of the items in the measurement scale. Visual comparison of response distributions for the eight items enables the relative strengths and weaknesses of the educational technology use to be determined at a glance.

Survey instruments are often designed to contain purely quantitative (Likert) scales, however, we advocate strongly for the use of the qualitative questions in the instrument, to increase the explanatory power of the instrument with respect to the evaluation of technology use (in line with Leavy, 2017). The qualitative component of the survey was able to explain how increased confidence impacted upon affective perceptions, the structure of the course increased perceptions of design, the practicality of the outcomes contributed to perceptions of learning, the interface of the course enhanced learner satisfaction, the clear instruction strengthened understanding of concepts, the flexibility of the course enabled self-directed learning, and the course enabled students to immerse in the learning environment. Without the qualitative components of the survey, and the corresponding analysis that took place, it would not be possible to understand the reasons for people’s evaluations of the technology use. This is in broad accordance with arguments for greater use of mixed methods research from elsewhere in the educational research field (Creswell, 2015; Han, 2020; Scoles et al., 2014).

For the open-ended questions “*Are there any ways that this survey could be improved”,* participants were generally satisfied with the survey and believed that it was a comprehensive survey considering all relevant domains. The survey was also considered to be well-structured and well-designed. The survey questions were generally perceived as being easy to understand and straightforward. Participants made five suggestions for the questionnaire items, including improving the wordings, improving the layout, reducing the number of questions, etc. Consequently, adjustments have been made to the items in our final instrument, and at other times considerations for future implementations are proposed. Regarding the wordings of the questions, in the process of achieving the best model fit, we removed all negatively-worded items in our finalized model, so that all items were positively-worded (as per participant suggestions). Researchers have argued that positively-worded items and the negatively-worded items may not be measuring the same underlying trait (Onwuegbuzie & Daniel, 2002), and suggest that using mixed stems (i.e., positively- and negatively-worded items) may reduce score reliability. Hence we do not see the shift to only including positively phrased questions as a limitation of the final instrument.

In response to the participants’ thoughts about providing in-depth understanding of different aspects in the survey, open-ended questions asking about each of the eight dimensions could be included in the questionnaire. This would need to be considered with respect to the extra time it would take to complete the survey. In regard to the feedback about the layout of the questionnaire, researchers could add graphics and images to the online instrument, use toggle buttons throughout the survey, or only show 10 questions per page. Regarding the length of the questionnaire, participants would like to make it shorter with fewer questions. The model reduction process resulted in a 28-item survey as opposed to 38 items, addressing this concern.

Furthermore, regarding the number of response options in the questionnaire, participants suggested a wide range of options, from 0-point up to 10-point scale. Nevertheless, researchers claim that the 7-point scale provides more variety of options than five items, which in turn increase the probability of accurately reflecting respondent perceptions (Joshi et al., 2015). Hence items in the CEUTIE model are measured on a 7-point Likert scale (0 = strongly disagree to 6 = strongly agree). Whereas for 10-point scale there are criticisms about the difficulty to clearly state the word labels in the scale, and it would be more time consuming for respondents to process the answers (Darbyshire & McDonald, 2004).

The qualitative responses of the participants brought up an interesting point – to what extent does an evaluation instrument need to be context-specific? The attempt in this study has been to derive a general instrument based on broad themes evaluated in the educational technology field, to enable somewhat objective and standardized comparison. However, there may be circumstances under which technology and context-specific factors are of interest, which may result in the adaption of the CEUTIE instrument or the use of other instruments. While we used the wording “*Coursera platform*” for our particular study context, this phrase could be replaced by any other learning technology being evaluated in other studies (see Table 6 in Appendix for the final instrument). This leads to a potential limitation of this study – we only validated the instrument in a single context, that of an open online course on *Excel for Business: Essentials* hosted on the Coursera platform. As well, we view this short-term limitation of a single validation context as potentially forming part of a long-term opportunity relating to our research. Because the instrument is designed to be comprehensive and generic, future research can include a variety of learning contexts and environments.

A range of stakeholders including researchers, educators, institutions, policy-makers, and government organizations can use this instrument to perform comprehensive comparative evaluations of different technologies (e.g. virtual reality, mobile learning, digital books, Google Docs, Scratch, gamification, etc.) used in different contexts (e.g. demographic, geographic, cultural, pedagogical). This capacity to accurately and consistently contrast uses of technology in education across different contexts can in turn support better understanding of technology use across the field.

When EFA was applied the large majority of items seemed to clump around one super concept of technology use in education. On the one hand, this indicates a degree of reliability for the instrument overall, in terms of it achieving a goal of evaluating the construct of technology use in education. On the other hand, the factor structure of the final model did not statistically emerge from the EFA analysis. However, there were theoretical grounds for the factor structure used in the CFA, based on previous empirical analysis of factors relating to the evaluation of technology use in education, and it is this factor structure that has been validated using the CFA analysis. That is to say, there are strong theoretical reasons for using the CEUTIE model, as well as practical utility in terms of providing insights into different aspects of technology use in education. Future work could further examine the CEUTIE model to determine its validity using both CFA and EFA analysis in different contexts.

## Appendix

Tables 4, 5 and 6

**Table 4:** Participant thoughts about the Coursera *Excel Skills for Business: Essentials* course

Categories	Examples	Number of references
Thoughts about the Coursera MOOC		2046
Affective Elements	• It was great learning excel through Coursera! • Increased my motivation towards excel • Really learn in a way I can feel confident about my skills in excel something I didn't have before • It is my personal opinion as I sometimes lost interest a bit	448
Design	• The course material is comprehensive and detailed • The weekly assessment also provides a current understanding of one's level at the moment • I liked the format of the course, how it was divided into weeks and how each week had the same structure and how the videos were separated so that I wasn’t watching the all the videos and then doing the other activities. The structure of the course helped me learn • It was very clear what each week would be about and what materials were relevant for which sections • Sometimes the assessment questions didn't match the course taken on that week • Certain assessment questions were not very clear • It would be better if you explain all ribbon briefly first of all and then start the course	363
Learning Outcomes	• I applied this knowledge in practical way. This course taught me the importance of Excel in professional life • Hey before joining the Coursera I didn't know how to use the excel and now I'm expert in the excel • I had 0% knowledge about excel when I first entered into the course. But now I think my skills on this have developed much • But the questions form was a bit difficult for me because I'm not good at English so there were some questions I couldn't understand it	325
Technology	• The platform is easy to use • The user interface of the course was very helpful • I had no technical difficulties using it • Some of the connection has a problem several times and the page came out very slowly	238
Teaching/Pedagogy	• The course instructor is fully prepared in which course was explained in a clear and easy to understand manner • The teaching was highly engaging, lively and top-notched • Particularly loved the instructors for their cheerful and motivating demeanor • If you can be more explicit when teaching and bring in more examples or illustrations it will be very better	163
Behavior	• Flexibility to decide for myself on the subjects I need some more practice or not • Learning Excel skills through Coursera enabled me to complete the tasks at my own pace while keeping me on track by using the deadlines as a guide • No specific interaction with other students for this course • I did not get a chance to interact with other students in the course	84
Institutional Environment	• Am particularly from Kenya and was not in a position to pay for the course but I benefitted from the financial aid • This is the first time I followed an IT course online. Thanks a lot for Coursera providing this valuable course for free for me who has poor economic condition • The only concern that I have is that it is pretty difficult to contact with the tutor of this course. If you have any question you cannot find any help. The forum does not help at all. I do not have any email contact or something like that if I need help • I think Coursera should make it free for the students who are from the third world	45
Presence/Community	• It really connects me and makes me immerse in this learning environment • I don't find there to be any sense of “community” • I do not particularly look forward to being a part of a community when I'm learning something so even though I disagreed to not connecting with students, it's simply because I do not want to • I did not get the questions asking participation being part of a community	21
None/NA		359

**Table 5:** Participant comments about the survey instrument

Categories	Examples (Codes)	Number of references
Positive aspects of the survey		**445**
• Good survey	• Survey was good • Nice survey • the survey is already a good one • The survey was good. I enjoyed filling it	237
• Nothing needs to be improved	• No improvements needed • It was really good and no need for improvements • The survey looks quite good to me and no changes are needed • I found it perfect. So, I don't think there are any other suggestions from my side • No need to improve, it's good already	144
• Comprehensive survey	• The survey was already able to cover all the necessary aspects • I think this survey covers up all the topics that appear on Coursera • The survey was comprehensive • The survey is exhaustive and complete all relevant points	57
• Well designed, well structured	• The survey is very well designed, no issues found • It was well structured • The survey was well designed • I think this survey is in it's the best state and would be very helpful to get the exact response	21
• Easy to understand, direct questions	• The survey is short and easy to understand • The survey was very easy to understand and answer • The survey content is good enough and it contains appropriate questions • The questions are asked directly	16
Improvement of the survey		**414**
• Wordings of the questions	• I think the mixture of negative–positive statements is strategically placed but not all are proficient in English, so, it might be confusing or mixed up by others • It did better put more explanation on the survey • For a non-native English speaker, some of the affirmations may be a bit tricky/confusing • Questions could be worded in a simpler matter to increase understanding of what was being asked	118
• In-depth understanding of different aspects	• Probably discuss more Coursera specific features • How the participants are using the knowledge of this course can also be a part of this survey • The survey could incorporate more questions about the interaction (type, quality, frequency) between people • Allow participants to share more of the challenges	62
• The layout of the questionnaire, better visual presentation	• It will be improved by adding some graphical and image structure. It will look attractive • The up-down arrows are somehow irritating. Can use toggle button instead • This is good maybe color change here and there • It is better to divide the questions into more than one page	49
• Make it shorter, fewer questions	• I think the questions are too many and some of them look similar, so they can be omitted to reduce the survey time • Fewer questions will be more effective • Although, I feel it is too long to be on a page and the questions can be grouped • Too many questions. Reduce the number of questions if possible	43
• Answers options/rating scales	• Include more questions where the users can provide feedback in the form of text answers (you can keep them optional in case someone doesn't wish to provide a textual answer/feedback) • And also there could be questions for each week separately so that learner could tell how much he is impressed with the individual week or module • Use the scaling method of 0–10 • It would be better if there were fewer options for answering all the questions, instead of 5/6 options it could be 3/4	42

**Table 6:** Final 28 item “Comprehensive Evaluation of Use of Technology in Education” (CEUTIE) instrument

Learning Outcomes (LO)
The way [the technology] was used in this course helped me to learn more about the subject
The way [the technology] was used in this course helped me to perform better in this subject
The way [the technology] was used in this course increased my skills in the subject area
The way [the technology] was used in this course has improved my level of knowledge in the subject area
Affective Elements (AE)
The way [the technology] was used in this course enhanced my attitudes towards the subject
The way [the technology] was used in this course helped to improve my confidence in this subject
The way [the technology] was used in this course enhanced my motivation to learn
The way [the technology] was used in this course made learning more enjoyable
Behavior (B)
The way [the technology] was used in this course increased my participation
The way [the technology] was used in this course increased my ability to reflect upon my learning
The way [the technology] was used in this course enhanced my overall engagement
Design (D)
The way [the technology] was used in this course enhanced the overall design of the subject
The way [the technology] was used in this course enhanced the subject content
The way [the technology] was used enhanced the course structure
Technology (T)
[The technology] used in this course was of high quality
The functionality of [the technology] used in this course helped me to learn the subject
[The technology] used for learning in this course was easy to use
[The technology] used for learning in this course was reliable
[The technology] was useful to support learning in this course
Teaching/Pedagogy (TP)
The way [the technology] was used in this course increased my overall perceptions of the teaching quality
The way [the technology] was used in this course enhanced teaching
The way [the technology] was used in this course enhanced feedback processes
Presence/Community (PC)
The way [the technology] was used in this course enhanced my sense of connection with the teacher
The way [the technology] was used in this course enhanced my sense of being present in the class
The way [the technology] was used in this course enhanced my sense of connection with other students
Institutional Environment (IE)
There was good technical support for the use of [the technology] in this course
The institutional support provided by Coursera Inc for the use of [the technology] positively contributed to my learning experience
The institution (Coursera Inc) embraces the use of [the technology] in education

Please add any comments to clarify your responses or add any other thoughts about using [the technology] in your course._________________________________________________________________________
